# Supplementary material for: Prevalence of Plastic and Hardware Foreign Bodies among Goats at Malawi Markets
Source: Animals (Basel). 2024 Jan 1;14(1):147. doi: 10.3390/ani14010147 (PMC10778286; doi:10.3390/ani14010147)
Supplement: Supplementary file 1 [file animals-14-00147-s001.zip › 2023_ButcherSurveyManuscript_SupplementaryMaterials.docx]

Supplementary Materials for

“Prevalence of plastic and hardware foreign bodies among goats at Malawi markets”

**Authors:** Jonathan H.I. Tinsley^1^, Andrews C.L. Safalaoh^2^, Winchester Mvula^2^, Javier Ventura-Cordero^1^, Taro Takahashi^3^, Patson Nalivata^2^, Jan A. Van Wyk^4^, Eric R. Morgan^1^, Paul M. Airs^1^

**Affiliations:**

1. Biological Sciences, Queen’s University of Belfast, 19 Chlorine Gardens, Belfast, BT9 5DL, UK

2. Animal Science Department, Lilongwe University of Agriculture and Natural Resources (LUANAR), P.O. Box 219, Lilongwe, Malawi

3. Agri-Food and Biosciences Institute, Hillsborough, Co. Down, Northern Ireland BT16 6DR, UK

4. Department of Veterinary Tropical Diseases, University of Pretoria, Private Bag X20, Pretoria, South Africa

**Table S1. Base demographics of respondents and areas surveyed per district**

| **District** | **Responses** | **Sex**  **(Male : Female)** | **Average years of experience** | **Rural town** | **Village** | **Town** |
| --- | --- | --- | --- | --- | --- | --- |
| **Chitipa** | 18 | 18:0 | 9 | 7 | 9 | 2 |
| **Lilongwe** | 50 | 50:0 | 9 | 33 | 14 | 2 |
| **Nsanje** | 50 | 50:0 | 2 | 25 | 10 | 15 |
| **Salima** | 20 | 20:0 | 14 | 13 | 4 | 2 |
| **Thyolo** | 12 | 12:0 | 19 | 2 | 9 | 1 |
| ***Total*** | *150* | *150:0* | *10* | *80* | *46* | *22* |

**Table S2. Sourcing of goats by district and distance travelled to market**. Butchers were asked to list local locations and distant locations where goats were purchased from. Responses were tallied based on the presence of local and distant locations references. Importance of distance and knowledge if stolen were multiple choice responses from ‘very’ = ‘very important’, ‘less’ = less important, and ‘not’ = not important.

| **District** | **Responses** | **Goat sourcing**  **location relative to the market** | | | **Importance of**  **‘goat distance from farm’**  **for purchase** | | | **Importance of**  **‘knowledge if goat stolen’**  **for purchase** | | |
| --- | --- | --- | --- | --- | --- | --- | --- | --- | --- | --- |
|  |  | **All**  **local** | **Mixed** | **All distant** | **Very** | **Less** | **Not** | **Very** | **Less** | **Not** |
| **Chitipa** | 18 | 6 | 12 | 0 | 1 | 10 | 7 | 18 | 0 | 0 |
| **Lilongwe** | 50 | 41 | 9 | 0 | 0 | 46 | 3 | 0 | 47 | 2 |
| **Nsanje** | 50 | 14 | 25 | 11 | 14 | 15 | 20 | 50 | 0 | 0 |
| **Salima** | 19 | 11 | 8 | 0 | 10 | 0 | 9 | 19 | 0 | 0 |
| **Thyolo** | 12 | 1 | 11 | 0 | 0 | 3 | 9 | 12 | 0 | 0 |
| ***Total*** | *149* | *73* | *65* | *11* | *25* | *74* | *48* | *99* | *47* | *2* |

**Table S3. Plastic and hardware IFB prevalence among market butchers in different Malawi districts**

| **Type** | ***n*** | ***n***  **goats *** | **Plastic IFBs** | | | | **Hardware IFBs** | | | |
| --- | --- | --- | --- | --- | --- | --- | --- | --- | --- | --- |
|  |  |  | **Considered a problem**** | **Plastic in the last 5 goats…†** | | | **Considered a problem**** | **Hardware in the last 5 goats…†** | | |
|  |  |  |  | **Any** | **Ave** | **Total** |  | **Any** | **Ave** | **Total** |
| **City / Town** | 22 | 110 | 10 (45.5%) | 10 (45.5%) | 0.9 | 19 (17.3%) | 3 (13.6%) | 3 (13.6%) | 0.2 | 5 (4.5%) |
| **Rural town** | 80 | 400 | 76 (95%) | 49 (61.3%) | 1.5 | 121 (30.3%) | 54 (67.5%) | 15 (18.8%) | 0.5 | 15 (3.8%) |
| **Village** | 46 | 230 | 33 (71.7%) | 25 (54.3%) | 1.1 | 51 (22.2%) | 10 (21.7%) | 7 (15.2%) | 0.2 | 7 (3%) |
| ***Total*** | *148* | *740* | *119 (80.4%)* | *85 (56.7%)* | *1.3* | *191 (25.8%)* | *68 (45.3%)* | *38 (25.7%)* | *0.4* | *55 (7.3%)* |

* Derived from the 'last 5 goats' question, multiplying respondents by 5. ** Derived from the is plastic or hardware a problem question, shown as those who responded yes. † 'Any' answers >0 per respondent, 'Ave' = the mean from respondents, 'Total' = the tally of all goats.


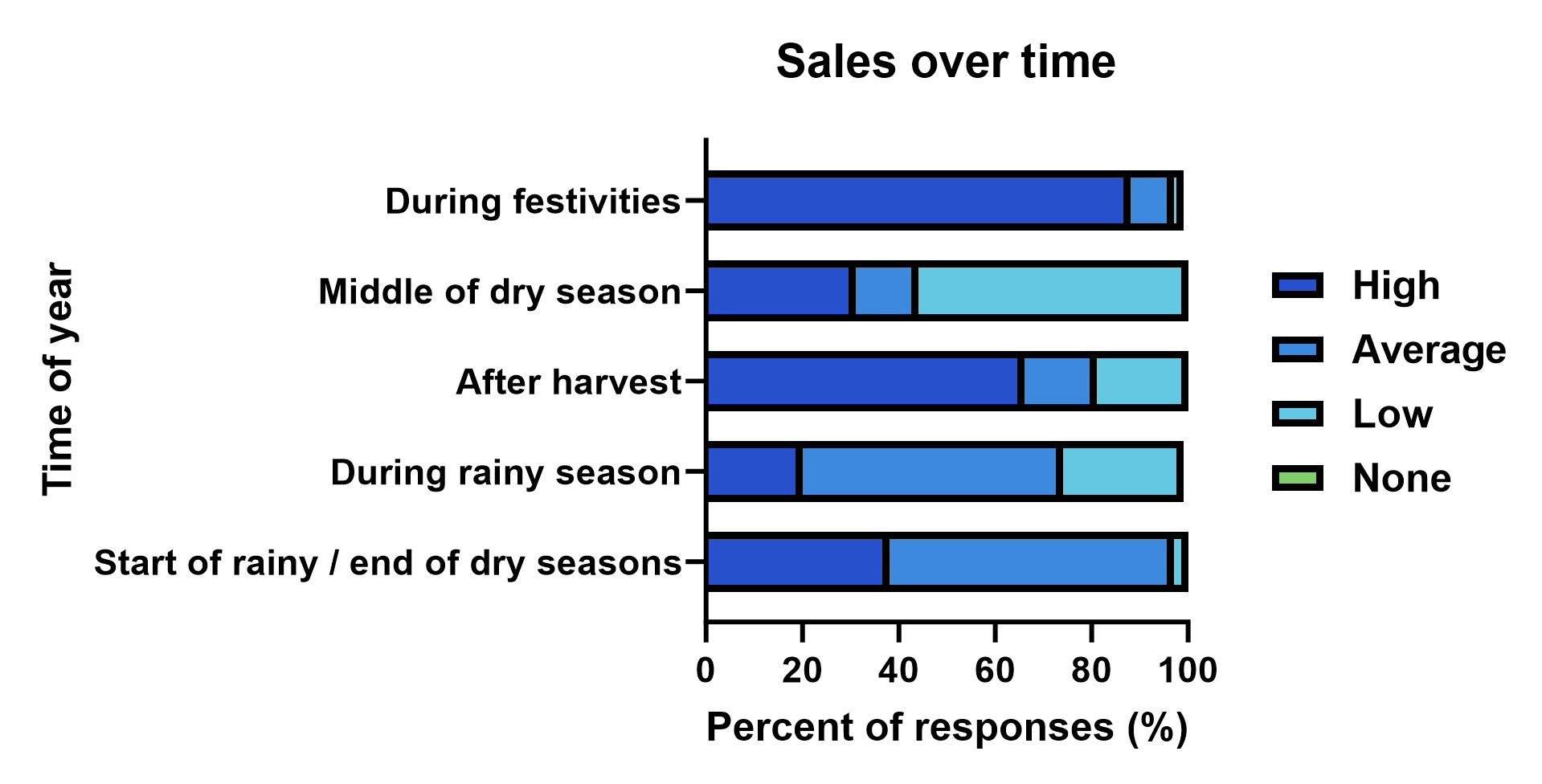


**Figure S1. Seasonal goat sales rank across study group.** Butchers were asked “how do you rate the frequency of goats slaughtered during the following periods?” N=103 across all districts.

**File S1**. Anonymous raw questionnaire responses and validation survey responses.

[File attached separately]
